# Ego depletion and implicit and explicit determinants of condom use intentions: an experimental study among young men

**DOI:** 10.12688/openreseurope.15433.1

**Published:** 2023-01-18

**Authors:** Kenny Wolfs, Arjan E.R. Bos, Fraukje E.F. Mevissen, Hugo Alberts, Jacques J.D.M. van Lankveld

**Affiliations:** 1 Faculty of Psychology, Open University of the Netherlands, Heerlen, The Netherlands; 2 Faculty of Psychology and Neuroscience, Maastricht University, Maastricht, The Netherlands; 3 Positivepsychology.com, Maastricht, The Netherlands

**Keywords:** condom use; ego depletion, implicit attitudes, dual-process model

## Abstract

Background: The Reflective Impulsive Model of Strack and Deutsch (2004) is a dual-process model and could be a dynamic theoretical framework of sexual risk behavior that is able to predict condom use under different circumstances. If we apply the Reflective Impulsive Model to sexual risk behavior, implicit attitudes regarding sexual risk behavior should have a stronger impact on behavior when working memory capacity is low. Explicit attitudes have a strong impact on intentions, which diminishes as participants have less working memory capacity.

Methods: In this study, we induced a state of ego depletion to examine the impact of low working memory capacity on implicit and explicit attitudes and condom use intentions. Young, male participants ( *N* = 66) were randomly assigned to either an ego depletion condition (difficult calculus task) or a placebo condition (easy calculus task). At baseline, a questionnaire measuring explicit attitudes and intentions to use a condom, and an Implicit Association Test measuring implicit attitudes towards condoms were administered. After the ego calculus task, participants once more completed the questionnaire and Implicit Association Test.

Results: We found no evidence that ego depletion had an effect on intentions to use a condom in young men. Explicit attitudes predicted intentions to use a condom, regardless of participants’ state. We found no relationship between implicit condom attitudes and intentions to use a condom, neither in the ego depletion nor in the placebo condition.

Conclusions: The implications of this null finding are discussed.

## Introduction

Globally, there were 1.5 million new HIV infections in 2020, most of which were transmitted through unsafe sex ( UNAIDS, 2020). In order to develop safe sex interventions, it is important to explore the determinants of condom use ( Bartholomew *et al*., 2001; Eldredge *et al*., 2016). Although condom use during sexual encounters is essentially a dyadic process, most research on condom use determinants has focused on individual factors (but see: Starks *et al*., 2018; Svenson *et al*., 2002). Contextual influences, that are only present in certain situations, are important individual-level predictors of sexual risk behavior. Two important contextual influences on condom use are alcohol intoxication ( Cooper, 2002; Leigh, 2002; MacDonald *et al*., 2000a; Norris *et al*., 2013; Norris *et al*., 2009; Zawacki, 2011) and sexual arousal ( Ariely & Loewenstein, 2006; George *et al*., 2009; MacDonald *et al*., 2000b; Norris *et al*., 2009; Skakoon-Sparling *et al*., 2016). In order to more accurately predict sexual risk behavior, dynamic models of sexual risk taking are needed which take contextual influences such as alcohol or sexual arousal into account and can explain how alcohol and sexual arousal cause this shift in behavior.

Dual-process models ( Evans, 2003) may provide a dynamic theoretical framework to investigate contextual factors in condom use intentions. They postulate two different systems that operate simultaneously and jointly determine behavior. The explicit system encompasses one’s knowledge, explicit attitudes, and risk assessments related to (un)safe sexual behavior. This is a slow process requiring ample cognitive resources. On the other hand, the implicit system encompasses one’s implicit attitudes toward condoms and one’s impulses to engage in unprotected sex. This system works rapidly and functions automatically, in the sense that it does not require large cognitive resources. This implies that when cognitive resources are sufficiently available, the explicit system will exert its influence on the behavior that is performed. It will be able to overrule the implicit system in case of conflict between both systems. If cognitive resources are insufficient, however, the reflective system will no longer be able to override the impulses from the implicit system and the implicit system will more strongly determine behavior ( Evans, 2003).

The Reflective Impulsive Model (RIM) of Strack and Deutsch (2004) is an application of the aforementioned dual-process models, and might be able to explain the effect of alcohol use and sexual arousal on condom use. Working memory capacity plays an important role in the Reflective Impulsive Model. This capacity is reduced when individuals are sexually aroused ( Carvalho *et al*., 2011; van Lankveld & Smulders, 2008), because sexual stimuli are salient and automatically capture one’s attention. Alcohol is also known to reduce working memory capacity ( Boha *et al*., 2009; Molnár *et al*., 2009). Thus, when individuals are under the influence of alcohol or when they are sexually aroused, the RIM predicts that their intentions to use a condom will be less determined by their explicit cognitions, such as their positive attitudes towards condom use or their risk assessment of contracting a sexually transmitted infection. In these circumstances, their condom use intentions will to a larger extent be determined by their implicit attitudes towards using condoms. Therefore, if their implicit attitudes towards condoms are less positive than their explicit attitudes towards condoms, individuals will be less inclined to use a condom when they are in a sexual situation when their working memory capacity is limited. This prediction has been tested in two experimental studies. In line with the RIM, the first study ( Wolfs *et al*., 2019) found that, when participants were not sexually aroused, their intentions to use a condom were exclusively predicted by their explicit attitudes toward condom use. However, when they were sexually aroused, their condom use intentions were predicted by both explicit and implicit attitudes. A second lab study ( Wolfs *et al*., 2022a) tested the impact of implicit and explicit attitudes towards condoms on condom use intentions, while both alcohol intoxication and sexual arousal were manipulated. The strongest effect of explicit attitudes on condom use intentions was found when participants were sober and not sexually aroused; this effect was smaller in the intoxicated and sexually aroused conditions. However, no main or interaction effects of implicit attitudes were found in this study, although both alcohol intoxication and sexual arousal were found to diminish working memory capacity.

In the current study, we aimed to test the impact of implicit and explicit attitudes towards condoms on condom use intentions, while manipulating working memory capacity without inducing alcohol intoxication or sexual arousal. The rationale for this decision is based on the multiple effects of sexual arousal over and above the effect on working memory capacity ( George *et al*., 2009; MacDonald *et al*., 2000b), and, likewise, the effects of alcohol intoxication ( Davis *et al*., 2014; Norris *et al*., 2009) on intentions to use a condom. These direct effects on condom use intentions imply that sexual arousal and alcohol intoxication are not pure manipulations of working memory capacity as they also directly affect one’s intentions to use a condom. We therefore aimed to use an experimental manipulation that reduces working memory capacity without directly affecting condom use intentions.

A way to experimentally diminish cognitive resources is by inducing a state of ego depletion. The term ego depletion comes from the limited strength model ( Muraven & Baumeister, 2000; Muraven *et al*., 1998). This model postulates that only a certain amount of mental resources are available, and once those resources are depleted, one will show decreased self-control performance. The phenomenon of being low on ‘self-control resources’ is known as *ego depletion*. A meta-analysis by Hagger *et al*. (2010) concluded that being in a state of ego depletion reduces one’s ability to exert self-control. Substantial effect sizes were also found for the negative effects of ego depletion on effort, negative affect and subjective fatigue ( Hagger *et al*., 2010). Among others, Hofmann *et al*. (2008) compared being in a state of ego depletion to a state of low working memory capacity. These authors even suggested that the current research into dual-process models builds directly on the earlier research into ego depletion and the cognitive load literature. Cognitive capacity, which is necessary for the reflective (explicit) system to function, is crucial in all dual-process models ( Evans, 2003). Ego depletion has an effect on executive functioning, including logical reasoning skills ( Schmeichel *et al*., 2003). Schmeichel (2006) claimed that performing an attention-dividing task has a negative effect on a subsequent working memory task. In another study Schmeichel (2007) found that working memory capacity was diminished after a task that depleted self-control. Thus, using an attention-control task ( Alberts *et al*., 2011) to induce ego depletion is expected to also impair working memory capacity.

Apart from having an effect on working memory capacity, being in a state of ego depletion has also been linked with heightened risk taking regarding finances ( Bruyneel *et al*., 2009), high caloric food ( Imhoff *et al*., 2014) or heightened risk taking in general. In the study by Imhoff *et al*. (2014), general risk taking was conceptualized as showing higher levels of sensation seeking and higher risk-tolerance in critical road traffic situations.

To our knowledge, there has been no previous study of the direct effect of ego depletion on sexual risk taking. It has been proposed by Schmeichel *et al*. (2010) that ego depletion increases an approach bias towards immediate gratification. Nolet *et al*. (2015) investigated the effect of ego depletion on sexual self-regulation and found that being in a state of ego depletion made it harder for participants to inhibit their sexual arousal, which is a strong predictor of sexual risk taking, as we mentioned earlier. Although a more recent meta-analysis ( Hagger *et al*., 2016) concluded that the ego depletion effect has been heavily overestimated, the effect sizes varied widely across the different areas in which it was studied, justifying the research on ego depletion in the current study.

An inevitable assumption in a dual-process model of sexual risk taking is that individuals are more negative towards condom use on the implicit level than on the explicit level. If the implicit attitudes have a stronger impact because working memory capacity is diminished, the shift in behavior away from condom use can only be accounted for if implicit attitudes are more negative than explicit attitudes. In one of our earlier studies on explaining sexual risk taking we found that implicit attitudes towards condom use were indeed more negative than explicit attitudes towards condom use (reference removed for peer review). We will test whether this finding is replicated in this study. Although dual-process models aim to predict condom use behavior, experimental study designs, yielding scientific evidence of causality, meet with practical and ethical problems when they include sexual behavior as a criterion variable. For this reason, the study of contextual influences on condom use predominantly employs condom use intentions as the best available proxy for actual condom use ( Tolstedt, 2001; Turchik & Gidycz, 2012).

In the current study we aimed to reduce working memory capacity through ego depletion. We predict that when participants are depleted, they will show weaker intentions to engage in safe sex (Hypothesis 1), shown by a main effect of condition on intentions to use a condom. Additionally, we will also test an application of the RIM ( Strack & Deutsch, 2004) to account for sexual risk taking. In the RIM, working memory capacity moderates the relative effect that the reflective and the impulsive system have on behavior. In our study, we will therefore investigate whether ego depletion moderates the effect of explicit and implicit attitudes on intentions to use a condom (Hypothesis 2). We expect to find a main effect of explicit attitudes on intentions to use a condom. We also expect to find an interaction effect of condition and explicit attitudes, as well as an interaction effect of condition and implicit attitudes, on intentions to use a condom. Specifically, when participants are in a state of ego depletion, we hypothesize that explicit attitudes will have a smaller effect on intentions (Hypothesis 3). The second interaction effect implies that implicit attitudes will also have predictive value regarding intentions to use a condom, but only when participants are ego depleted. We tested the following hypotheses: Participants will report weaker intentions to use a condom in the ego depletion condition compared to the placebo condition (H1). There is a main effect of explicit attitudes on intentions to use a condom: positive explicit condom use attitudes will be associated with stronger condom use intentions. There is also an interaction between condition and explicit attitudes on intentions to use a condom: the previous association will be stronger in the placebo condition, compared to the ego depletion condition (H2). There is an interaction between condition and implicit attitudes on intentions to use a condom: the association of positive implicit condom use attitudes and stronger condom use intentions will be stronger in the ego depletion condition, compared to the placebo condition (H3). On the implicit level, as measured by the Implicit Association Test (IAT), participants will report significantly more negative attitudes than on the explicit level, as measured using a questionnaire (H4). This assumption is crucial to explain the working mechanism of a dual-process model of sexual risk taking.

## Methods

### Participants

Participants were students who were recruited by KW using flyers at the campus of the Maastricht University in The Netherlands, and via a research panel including psychology students who are interested in joining studies, or have to participate as part of their studies, in studies performed by students and researchers from the psychology department. They could participate for either course credit or a 10 Euro monetary reward. Recruitment took place between January and May 2016. Inclusion criteria was: male gender, age 18 years or older, and sufficient command of the Dutch or English language to be able to answer the questionnaires. We only included male participants, in order to be able to compare the results with a previous male-only study on a dual-process model of sexual risk taking ( Wolfs *et al*., 2019). A total of 66 participants were included and randomly assigned to the placebo (N = 33) or the ego depletion condition (N = 33). Randomization was performed using the number generator Random, generating a random order of conditions while ensuring each condition was equally represented. Participant age ranged from 19 to 30 years with an average of 22.3 years ( *SD* = 2.63). This young age range was inherent to the student character of the study population. Twenty-seven participants were native Dutch speakers; the other 39 had other nationalities and participated in the English version of the study. Three participants identified themselves as gay, while the remaining 63 identified themselves as heterosexual. Twenty-five participants were in a relationship, 36 participants were single and five did not reveal their relationship status. All participants continued to the end of the experimental session.

### Procedure

Participants could choose to participate in either English or Dutch. Upon entering the laboratory, they read an information letter about topic and procedure of the study, then signed an informed consent form. The true purpose of the experiment was not revealed at this moment. Participants were told that the study aimed to investigate the effect of cognitive tasks on sexual behavior, reaction times and physical endurance.

**Figure 1.**
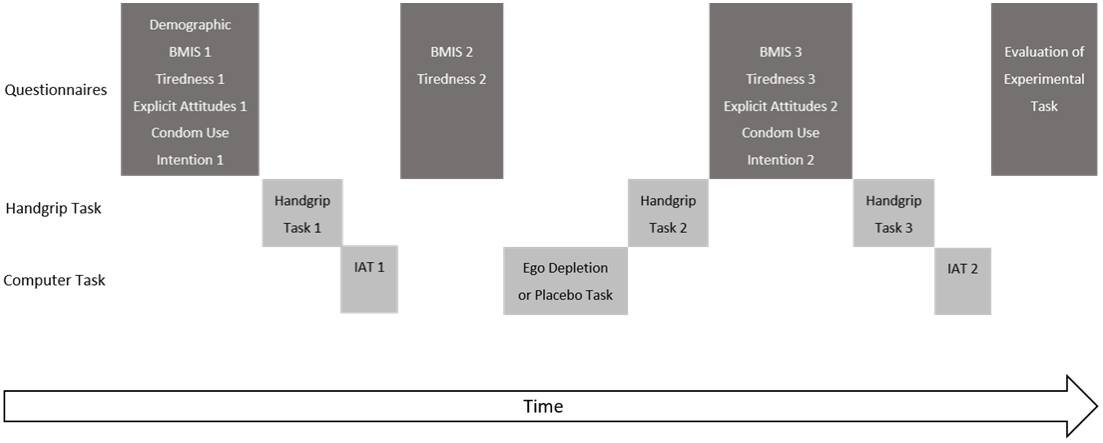
Experimental Flow.

A visual overview of the study flow is shown in Figure 1. Questionnaires were completed at four moments during the lab session. First, the baseline survey including demographic variables, explicit measures of condom attitudes, and intention to use a condom was administered. The baseline survey also included two manipulation checks, measures for mood and for tiredness. It was followed by a handgrip task. Baseline measurement ended with an IAT to measure implicit attitudes. After finishing the IAT, participants were asked how tired they were, and again completed the Brief Mood Introspection Scale (BMIS). These are standard checks to see if ego depletion has an effect on tiredness or mood ( Alberts *et al*., 2011), but considering that the IAT is a demanding task in itself and requires cognitive resources, we also used these measures throughout the protocol to check whether performing an IAT also had effects on tiredness and mood.

Subsequently, participants performed the calculus task. This task was either easy (placebo condition) or difficult (ego depletion condition. Next, participants did the handgrip task again and then completed a third package of questionnaires. This package again contained the BMIS, a rating of subjective tiredness, the explicit attitudes towards condom use as well as the intention to use a condom. After completing these questionnaires, participants performed the handgrip task once more, followed by the second IAT. Finally, participants answered questions regarding how boring, frustrating and tiresome the IAT and the calculus task were. At the end, participants were thanked for their participation, and debriefed.

### Experimental manipulation

To create an ego depletion state, an attention-control task including calculations was used. This and similar attention-control tasks had previously been used successfully in several studies on ego depletion ( Alberts *et al*., 2011; Alberts *et al*., 2007; Faber & Vohs, 2004; Schmeichel *et al*., 2003).

Participants allocated to the ego depletion condition performed a calculation task for eight minutes, while listening to a voice reading numbers out loud through headphones. In the placebo condition, participants were not distracted and did not wear headphones. The calculation task in the ego depletion condition was to summate two-digit numbers, whereas in the placebo condition participants summated one-digit numbers. The calculation task became increasingly difficult in both conditions as more and more numbers had to be summated. Participants were instructed to complete as many calculations as possible, while remaining accurate and avoid making mistakes. After eight minutes, the experimenter informed the participants they could stop the calculation task.

### Measures

**Mood.** To measure mood throughout the experiment, we used the Brief Mood Introspection Scale (BMIS). The BMIS consists of 16 adjectives representing eight mood states (happy, loving, calm, energetic, fearful/anxious, angry, tired and sad) using two adjectives for each mood state. The higher the total score, the more positive the mood of the participants. Participants reported how much they felt like a certain adjective on a 5-point scale going from 1 (definitely do not feel …) to 5 (definitely feel …). In the present study sample, Cronbach’s alpha for the BMIS was satisfactory (α = 0.85).

**Self-control**. We used a handgrip to test if the manipulation managed to reduce self-control. Keeping the handgrip squeezed is fatiguing and becomes even a little bit painful after a while. Therefore, it can be used as a measure of physical stamina and self-control analogous to the protocol used by Alberts *et al*. (2011) and Muraven *et al*. (1998). It is expected that participants in the ego depletion condition will show a significantly larger drop in how long they can hold on to the handgrip compared to the placebo group. Not being able to hold on longer to the handgrip task is interpreted as being in a state of ego depletion, because participants are exerting less self-control ( Alberts *et al*., 2011).

**Tiredness.** We used a Visual Analog Scale (VAS) to measure how tired participants felt. It consisted of a 10 mm line with two opposing statements at both ends. The two statements were “Not at all tired” or “Very tired.” It was expected that participants in the ego depletion condition would report a larger increase in tiredness, since the ego depletion task is expected to be more tiresome than the neutral task.

**Task rating.** We also checked whether participants would rate the calculus task differently in the neutral condition compared to the ego depletion condition. At the end of the experiment, participants were asked to rate how boring, interesting, annoying, frustrating. and exhausting they found the task on a 5-point scale. The same questionnaire was used in the study by Alberts *et al*. (2011). It was expected that participants would rate the calculus task as more boring, less interesting, more annoying, more frustrating, and more exhausting in the ego depletion condition. We also asked participants to rate the IAT using this same questionnaire. This allowed us to test if the IAT was also be perceived as ego depleting.

**Explicit condom use attitudes and intentions.** The explicit attitudes and intentions to use a condom were measured context-specifically. To accommodate both heterosexual and gay men, participants were first asked to read and then imagine a situation in which they met a new person in a bar. The encounter led up to the participant’s home and there was an opportunity to have sex. After imagining this situation, participants were asked to answer questions using a Visual Analog Scale (VAS).

Three items were used to measure *condom use attitudes*: “How wise would it be to use a condom in this situation? (Very unwise – Very wise)”, “How pleasant would it be to use a condom in this situation? (Very unpleasant – Very pleasant)”, “How hard would it be to use a condom in this situation? (Very hard – Very easy)”. These three dimensions of condom attitudes were based on dimensions of the UCLA Multidimensional Condom Attitude Scale (MCAS: Helweg-Larsen & Collins, 1994) and were used in previous studies ( Wolfs *et al*., 2019). A general attitude towards condom use was calculated by averaging the score on how smart, pleasant and easy participants thought it would be to use a condom in that situation. This attitude measure was also used in our previous study [blinded for review]. In the present study sample, the reliability was modest (Cronbach’s α = .61).

**Condom use intention**. Condom use intention was measured with one item: “How much trust do you have that you would use a condom in this situation? (Very little trust – A lot of trust).

**Implicit condom use attitudes.** Implicit condom attitudes were assessed using an Implicit Association Test ( Greenwald *et al*., 2003) consisting of five blocks. In the first block (18 trials), participants categorized words into two *attribute* categories (positive/negative) that were displayed in the left and right upper corners of the computer screen. The *positive* category was represented by the words “Gift”, “Peace” and “Health”, while the *negative* category was represented by the words “Hate”, “War” and “Disease.” In the second block (congruent practice, 36 trials), participants categorized words into the same attribute categories, while two *target* categories using pictures were also introduced. Target labels were “safe sex” (penetration with a condom) and “unsafe sex” (penetration without a condom). These categories were both represented by three pictures each either displaying vaginal penetration with or without a clearly visible condom. The setup of the third block (congruent test, 48 trials) was similar to the second block. In the fourth block (incongruent practice, 36 trials), the positions of the target categories (e.g. safe sex/unsafe sex) in the upper corners of the screen were swapped. In order to categorize words, participants now had to press the opposite keys of the ones they had to use in blocks one to three. The positions of “safe sex” and “unsafe sex” remained the same. The fifth (incongruent test) block (48 trials) had the same setup as the fourth block. The D600-score was calculated according to the recommendations of Greenwald *et al*. (2003).

### Statistical analyses

Statistical analyses were performed using SPSS Version 24 ( IBM Corp., 2016). The same analyses can be performed using open access software ( The jamovi project, 2022). To achieve a power of 0.80 with a medium effect size ( *f* ^2^ = 0.15) with an error probability of 0.05, a total sample size of 66 was needed. Missing data were not imputed before analysis. First, we checked whether study language influenced outcomes using independent samples t-tests with language as between-subjects variable on relevant measures, such as condom attitudes and intentions to use a condom. The two groups did not differ significantly on age ( *t*(57) = .98; *p* = .38), implicit attitudes toward condom use ( *t*(57) = .42; *p* = .68), explicit attitudes towards condom use ( *t*(57) = -1.94; *p* = .07), or intention to use a condom ( *t*(57) = -1.55; *p* = .13) at baseline. Data of both study language subgroups were therefore pooled.

Firstly, we examined possible mood effects to test whether intentions were (partially) influenced by mood fluctuations throughout the experiment. A general effect of time was tested using a repeated measures ANOVA. Subsequently, we ran independent t-tests with difference scores on the BMIS compared to baseline as dependent variable, and condition as between-subjects factor. Secondly, to check if the ego-depletion manipulation was successful, we first calculated the difference score of handgrip performance at both time points. We then performed an independent samples t-test with condition as between-groups factor and the difference scores as a dependent measures. A third manipulation check was performed using the Tiredness VAS. We calculated difference scores of tiredness at each time point compared to baseline. We ran an independent samples t-test with these difference scores as dependent variable and condition as between-subjects factor.

To test the first hypothesis, stating that intentions to use a condom were weakened due to being in a state of ego depletion, an independent samples t-test was performed with condition as grouping variable and condom use intentions as dependent variable.

The next analysis tested the second and third hypothesis. The second hypothesis stated that explicit attitudes would predict intentions to use a condom in the neutral condition. This prediction is tested as a main effect of explicit attitudes on intentions to use a condom. The second hypothesis also stated that, when participants are in a state of ego depletion, their explicit attitudes will have a smaller effect on intentions to use a condom. This second part of the second hypothesis is represented by an interaction effect between the ego depletion condition and explicit attitudes on intentions to use a condom. The third hypothesis stated that implicit associations would only predict intentions to use a condom in a state of ego depletion. This statement was tested as the interaction effect between condition and implicit attitudes on intentions to use a condom. A regression analysis was performed to jointly test both hypotheses. In this regression model, condom use intention at time point three was chosen as the dependent variable, and condition, explicit attitudes, implicit attitudes, the interaction between condition and change in explicit attitudes, and the interaction between condition and change in implicit attitudes were entered as predictors.

To test the fourth hypothesis, whether implicit attitudes were more negative than explicit attitudes, scores on the VAS and the IAT were standardized. The scores on the VAS were first centered around zero, so that a zero score on both the implicit and the explicit attitudes represented a neutral attitude. Then, both scores on both types of attitudes were divided by their standard deviation to convert them to the same scale. A paired samples t-test was performed.

We used the STROBE case-control checklist when writing our report ( Von Elm *et al.,* 2007).

## Results

### Manipulation check

The groups did not differ regarding change of mood after the first IAT ( *t*(57) = -.12; *p* = 0.91), or after the ego depletion task ( *t*(57) = 1,46; *p* = 0.15). Differences in intentions to use a condom between the two groups can therefore not be explained by mood changes caused by the ego depletion task. There was a significant group difference in how long participants could hold on to the handgrip before and after the calculus task ( *t*(57) = 2.01; *p* < 0.05). Participants in the ego depletion condition showed a larger drop in number of seconds that they could hold on to the hand grip ( *M* = -19.50; *SD* = 39.61) as compared to those in the neutral condition ( *M* = -2.21; *SD* = 27.1). Overall, independent of condition, participants reported feeling more tired as the experiment progressed in both conditions ( *F*(1, 53) = 3.29; *p* = .04). Participants in the ego depletion condition reported a greater rise in tiredness after the first IAT compared to baseline ( *M* = 40.29; *SD* = 20.97) than participants in the neutral group ( *M* = 30.71; *SD* = 22.21; *t*(57) = -2.49; *p* = .02). However, after the calculus task there was no difference between both groups on how tired they were compared to their baseline level of tiredness ( *t*(57) = -1.11; *p* = 0.27).

Compared with the neutral condition, participants in the ego depletion condition found the calculus task to be more boring ( *t*(57) = -2.47; *p* = .02), less interesting ( *t*(57) = 2.67; *p* = 0.01), more annoying ( *t*(57) = -3.63; *p* < 0.01) and more frustrating ( *t*(57) = -2.25; *p* = 0.03). The groups did not differ on how exhausting they found the task ( *t*(57) = -1.22; *p* = 0.90). It should also be noted that participants in the ego depletion condition rated the IAT as equally boring ( *t*(30) = 0.64 *p* = .65) and frustrating ( *t*(30) = 1.35; *p* = 0.18), but more annoying ( *t*(30) = 2.13; *p* = .04) and exhausting ( *t*(30) = 2.37; *p* = .02) than the ego depletion task.

To summarize the results of the manipulation check, participants in the ego depletion experienced a greater drop in the time they could hold on to the handgrip than participants in the neutral group. A secondary manipulation check (exhaustion, tiredness after the calculus task) however could not corroborate this finding, but this measure is not standard in ego depletion research. Possible changes in dependent measures, therefore, cannot be explained by differences in mood after the calculus task between both groups. These results indicate that our manipulation was successful, as determined by the important parameters of self-control, also used in the ego depletion experiments by Alberts *et al*. (2011).

Effect of ego depletion on condom use intentions

Condom use intention did not differ between conditions ( *t*(55) = .548; *p* = 0.59). The results of the regression analysis to test H2 and H3 are shown in Table 1. Explicit attitudes significantly predicted condom use intentions (β = .73; *t*(50) = 5.56; *p* < .001), but this effect was not moderated by ego depletion (non-significant interaction effect: β = -.20; *t*(50) = -1.39; *p* = 0.17). Implicit associations did not predict intentions to use a condom, whether participants were in a state of ego depletion (β = .02; *t*(50) = 0.10; *p* = .92) or in a neutral state (β = .05; *t*(50) = 0.37; *p* = 0.72).

**Table 1.** The Linear Regression Model with Intention to Use a Condom as Dependent Variable and Condition, Implicit Attitudes, and Explicit Attitudes and Their Interactions as Predictors.

Predictor	B	Std. Error	β	t-value	p-value
Intercept	9.16	30.02		.31	.76
Condition	-1.02	6.56	-.02	-.16	.88
Explicit Attitudes	1.08	.12	.73	5.57	<.001*
Implicit Attitudes	4.03	11.03	.05	.38	.72
Condition * Explicit Attitudes	-.30	.211	-.20	-1.40	.17
Condition * Implicit Attitudes	.72	6.79	.02	.10	.92
Mood	-.38	.41	-.10	-.877	.40

Implicit attitudes about condoms were more negative than explicit attitudes ( *t* = 9.96; *p* < .001). Explicit attitude scores had an unstandardized mean of 68.28 (z = 1.16), indicating that participants were positive about condoms on an explicit level, whereas the implicit attitudes had an unstandardized mean of -.19 (z = -.63), indicating that participants had a negative attitude towards condoms on the implicit level.

## Discussion

In this study, in a sample of young men, we explored whether a dual-process model can predict risky sexual behavior. Several assumptions of a dual-process model of sexual risk behavior were tested. For this purpose we used ego depletion as a manipulation to reduce working memory capacity. We investigated the effect of ego depletion on intentions to use a condom, and used explicit and implicit attitudes to investigate a possible explanation of this change in condom use intentions. We found no evidence that being in a state of ego depletion weakened one’s intentions to use a condom, implying that the hypothesis in question must be rejected. However, explicit attitudes towards condom use significantly predicted intentions to use a condom, independent of being in a state of ego depletion or not, supporting the pertinent hypothesis. We found no predictive value of implicit attitudes towards young men’s condom use on condom use intentions, neither in the neutral nor in the ego depletion condition, refuting the pertinent hypothesis.

An interesting finding was that the implicit attitudes towards condom use were more negative than the explicit attitudes, supporting the pertinent hypothesis. This implies that, if a dual-process model of sexual risk behavior would turn out to be valid in other studies, this difference in implicit versus explicit attitudes could explain the shift in condom use intentions that occurs when one’s working memory capacity declines. We are aware that explicit and implicit attitudes are measured using two very different methods. However, by using a visual analog scale with a neutral center point, we were able to test this hypothesis. Although the problem of using two different methodologies for two very different concepts is unavoidable, we believe that we were able to validly investigate the difference between implicit and explicit attitudes regarding sexual risk behavior.

The lack of a relationship between implicit attitudes and intentions to use a condom may speculatively be explained by the use of a calculation task to manipulate working memory capacity. Our manipulation check, the handgrip task, one of the most common manipulation checks in ego depletion research ( Hagger *et al*., 2010), showed that participants in both conditions were unable to hold on as long as they did at baseline, but the drop in performance was significantly larger in the ego depletion condition. An additional manipulation check on the level of fatigue, however, did not show a significant difference between the ego-depleted condition vs. the neutral condition. This measure was, however, not used in previous studies on ego depletion. We thus found only partial evidence for a successful manipulation, suggesting that the calculus task may not have been strong enough, even though it has been used repeatedly in previous ego depletion research (e.g., Alberts *et al*., 2011).

An alternative, and probably more plausible explanation for the lack of effect of ego depletion on condom use intentions or on the relationship between implicit attitudes and condom use intentions may be related to the two major limitations of this study. First of all, participants may have already been fatigued before they started performing the ego depletion task, due to the IAT preceding the ego depletion task. Second, the ego depletion concept has met with substantial critique in recent years, raising the question if it was the optimal manipulation to test a dual-process model. We elaborate on these two limitations in the following paragraphs.

The first limitation was that participants might already be ego depleted before performing the ego depletion task. Before the ego depletion task started, participants performed an Implicit Association Test. Participants in the ego depletion condition rated the IAT as equally frustrating and boring as the ego depletion task. It is therefore also possible that the IAT already put participants in a state of ego depletion, implying that the difference between the ego depletion and placebo condition may have been smaller than intended. Participants reported that they became more tired during the course of the experiment, suggesting that performing two IATs might indeed be cognitively exhausting. Future research should avoid using an IAT before an ego depletion task and only use it afterwards. One might argue that it could be best to abandon the IAT altogether in ego depletion research. Studies have found an effect of ego depletion on approach motivation ( Crowell *et al*., 2014; Schmeichel *et al*., 2010). This means that if one is in a state of ego depletion, one is more motivated to approach, for example, low-stakes betting ( Crowell *et al*., 2014).

To translate this approach behavior into an implicit measure, an approach-avoidance task might best be used instead of the IAT. Simons *et al*. (2016), for example, used an approach-avoidance task focusing on sexual risk behavior. In this experiment, however, we used an IAT, because we intended to measure implicit attitudes rather than implicit approach behavior.

The second limitation relates to the observation that ego depletion as a psychological construct has received considerable criticism lately. The original limited-strength model by Muraven *et al*. (1998) postulated that individuals possess a limited amount of cognitive and psychological resources that, once drained, will inhibit self-control. Carter and McCullough (2013), in their critique on ego depletion as an experimental manipulation, state that the ego depletion effect is being overestimated. They claim that the effect of ego depletion can be explained by a computational *opportunity cost model*. The second task, the outcome measure, is often seen by participants as irrelevant compared to the ego depletion task, and therefore the costs of showing self-control may outweigh the benefit. Carter and McCullough (2014) claim that a strong external motivator, such as money, can abolish the effect of ego depletion, because the cost of showing self-control after performing a task that required substantial mental effort (which is seen as another cost) is less than the benefit. Boucher and Kofos (2012) actually showed that monetary reward can indeed counteract the effects of ego depletion, supporting Carter and McCullough’s criticism of the limited-strength model. In our study, participants might have had a motivation to perform well on the ego depletion task, as well as on the IAT, which might explain why ego depletion had no effects on the implicit measure. Lately, some critique has also been voiced on ego depletion as an experimental manipulation. Recent pre-registered multilab experiments found no ( Hagger *et al*., 2016), or only a small (though significant) effect of ego depletion ( Dang *et al*., 2021). Critique on the manipulation in itself was also given in both older ( Hagger *et al*., 2010) and more recent meta-analyses ( Dang, 2018). Dang (2018) found that the two most effective ways to induce a state of ego depletion were working memory tasks and emotional videos. This suggests that ego depletion and working memory are related.

The question remains whether ego depletion (and, more specifically, the current task) was the best manipulation to test a dual-process model of sexual risk taking. We recommend using other, contextual factors that influence sexual risk taking such as alcohol intoxication ( Leigh, 2002; MacDonald *et al*., 2000a) or sexual arousal induction ( Ariely & Loewenstein, 2006; George *et al*., 2009).

From these limitations we can derive recommendations for future research. Firstly, we deliberately chose a manipulation that was not known to directly influence intentions to use a condom, instead of, for example, alcohol ( Cooper, 2002; George *et al*., 2009) or sexual arousal ( Ariely & Loewenstein, 2006; George *et al*., 2009). Since both alcohol and sexual arousal have also been shown to reduce working memory capacity ( Boha *et al*., 2009; Carvalho *et al*., 2011; Molnár *et al*., 2009; van Lankveld & Smulders, 2008), they might be more efficient manipulations to test a dual-process model of sexual risk taking. Secondly, future research should critically assess the number of IAT’s used in a research protocol when investigating the effect of ego depletion on implicit measures. Finally, should this experiment be replicated, a more homogenous group of participants regarding native language or sexual orientation should be examined. In conclusion, even though our experiment could not find conclusive evidence for the existence of a dual-process model of sexual risk taking, we do believe future research is necessary to either validate or discard this model of sexual risk taking. However, future research should deploy other experimental manipulations than ego depletion.

## Ethics and consent

The study was approved by the Ethics Review Board of the Faculty of Psychology of Maastricht University, Netherlands (ECP kenmerk 0212_2014).

All participants signed an informed consent form.

## Data Availability

Open Science Framework: Ego Depletion and Condom Use Intentions https://doi.org/10.17605/OSF.IO/78VWU ( Wolfs *et al*., 2022b) This project contains the following underlying data: - Labstudie 3 - Mixed Regression.sav - Labstudie 3 - Ruwe data.sav Data are available under the terms of the Creative Commons Zero "No rights reserved" data waiver (CC0 1.0 Public domain dedication).
