# Supplementary material for: Ego depletion and implicit and explicit determinants of condom use intentions: an experimental study among young men
Source: Open Res Eur. 2023 Jan 18;3:11. [Version 1] doi: 10.12688/openreseurope.15433.1 (PMC10445834; doi:10.12688/openreseurope.15433.1)
Supplement: Peer review history

# Reviewer report: Reviewer response for version 1

**DOI:** 10.21956/openreseurope.16684.r31495

**Published:** 2023-06-06

**Authors:** Maria-João Alvarez

**Affiliations:** 1 Faculdade de Psicologia, Universidade de Lisboa, Lisbon, Lisbon, Portugal

This is a very interesting study that helps us to more effectively understand the meaning of the “heat of the moment” as a state that seems to (negatively) affect condom use. In other words, people have the intention to use condoms but this seems to be “easily” sidelined by the intervention of several perceptual and contextual factors. One possible explanation resides in different explicit and implicit attitudes towards condom use and the relevance of these in behavioral intentions in various contexts.

 The study is very well described and, moreover, well thought out and resourceful, despite the several limitations that the authors themselves point out, which constitutes a further indicator that they are on the right track towards finding alternatives to answer the problem they raise: whether low working memory capacity can moderate the effects that implicit and explicit attitudes towards condom use have on the intention to use condoms.

 One of the main doubts that the study raises for me is the use of the Reflective Impulsive Model in the study of intention rather than of behavior. Dual-process models are studied in the context of behavioral actions and I am unaware of their use in the context of behavioral intentions. The authors refer to practical and ethical issues towards the inclusion of sexual behavior as a criterion variable, but alternatives ranging from the use of scenarios (e.g., Czopp *et al.*, 2004 ^1^) to the completion of narratives (e.g., Verplanken *et al.*, 1997 ^2^) could be employed.

 They also state that condom use intentions are the best available proxy for actual condom use and this has long been challenged by the intention-behavior gap (Sheeran & Webb, 2016 ^3^; Webb & Sheeran, 2006 ^4^) and by the enactment theories/self-regulatory theories (Armitage & Conner, 2000 ^5^; Mann *et al.*, 2013 ^6^). The studies the authors cite find a relationship with intention certainty or stability, but not with intentions as they are usually assessed.

 Another aspect that caused me surprise was the use of various "items" – adjectives – in the mood measure and in the task rating measure, three items in the attitudes measure and only one item for the outcome measure).

 The use of a single item implies limitations in terms of reliability, validity and sensitivity of the measure. In particular it is difficult to assess whether the individual's response is really an accurate representation of the construct in question or whether it is influenced by transient or random factors. However, sometimes asking the same thing in several ways may not bring any advantage. Therefore, it would be good to at least have an indication that in previous studies a single item behaved adequately.

 The authors assume, and find in a study they conducted, that individuals are more negative toward condom use on the implicit level than on the explicit level. However, there is research indicating that such attitudes may depend on the type of partner (regular vs. casual) (see Czopp *et al*., 2004 ^1^; Marsh *et al.*, 2001 ^7^), even though these studies made predictions in opposite directions, with Czopp predicting explicit attitudes more than implicit ones (vs. implicit rather than explicit in Marsh) on condom use in casual relationships. In the present study the explicit attitudes and intentions to use a condom were measured context-specifically, apparently within a casual or non-established relationship. Addressing the relational context would contribute to the discussion of the results found here.

 Lastly, the introduction should have a discussion of the use of the IAT for the assessment of attitudes towards condoms. In addition, the use of the IAT merits a reflection on the doubts raised toward this instrument in the assessment of attitudes towards condoms (see Czopp *et al.*, 2004 ^1^).

 Some questions of detail:

- In the background of the abstract there is talk of behaviors for implicit attitudes and behavioral intentions for explicit attitudes, not distinguishing behaviors from behavioral intentions.
- It is explained why the capacity of the working memory is reduced when individuals are sexually aroused. The reason(s) why this capacity is also reduced when alcohol is consumed should also be presented (page 3).
- Regarding the relationship of implicit attitudes and explicit attitudes towards sexual risk taking (page 4) other studies find implicit attitudes toward condom use to be more positive than negative (Czopp *et al.*, 2004 ^1^). I would elaborate some more on this relationship.
- As IAT is viewed as relatively impervious to an individual’s awareness of what is being measured it might be more appropriate that the order of completing measures be first the IAT and then the explicit measures on condom use. I think it might be worthwhile for the authors to justify their choice.
- Condom attitudes might not be highly accessible for participants who have not had sexual experience, and effects of implicit measures are expected for only highly accessible attitudes (see Marsh *et al.*, 2001 ^7^). If the authors have this sociodemographic data available, it should be included in the description of the participants.

If applicable, is the statistical analysis and its interpretation appropriate?

Yes

Is the study design appropriate and is the work technically sound?

Yes

Is the work clearly and accurately presented and does it engage with the current literature?

Yes

Are the conclusions drawn adequately supported by the results?

Yes

Are sufficient details of methods and analysis provided to allow replication by others?

Yes

Are all the source data and materials underlying the results available?

Yes

Reviewer Expertise:

Sexual risk behaviors; health education; behavior change interventions.

I confirm that I have read this submission and believe that I have an appropriate level of expertise to confirm that it is of an acceptable scientific standard.

## References

[1] : Implicit attitudes as potential protection from risky sex: Predicting condom use with the IAT. *Basic and Applied Social Psychology* .2004;26(2,3) :227-236 Reference source

[2] : Habit, information acquisition, and the process of making travel mode choices. *European Journal of Social Psychology* .1997;27(5) : 10.1002/(SICI)1099-0992(199709/10)27:5<539::AID-EJSP831>3.0.CO;2-A 539-560 10.1002/(SICI)1099-0992(199709/10)27:5<539::AID-EJSP831>3.0.CO;2-A

[3] : The Intention-Behavior Gap. *Social and Personality Psychology Compass* .2016;10(9) : 10.1111/spc3.12265 503-518 10.1111/spc3.12265

[4] : Does changing behavioral intentions engender behavior change? A meta-analysis of the experimental evidence. *Psychol Bull* .2006;132(2) : 10.1037/0033-2909.132.2.249 249-68 10.1037/0033-2909.132.2.249 16536643

[5] : Social cognition models and health behaviour: A structured review. *Psychology & Health* .2000;15(2) : 10.1080/08870440008400299 173-189 10.1080/08870440008400299

[6] : Self-regulation of health behavior: social psychological approaches to goal setting and goal striving. *Health Psychol* .2013;32(5) : 10.1037/a0028533 487-98 10.1037/a0028533 23646832

[7] : Heart versus reason in condom use: implicit versus explicit attitudinal predictors of sexual behavior. *Z Exp Psychol* .2001;48(2) : 10.1026//0949-3946.48.2.161 161-75 10.1026//0949-3946.48.2.161 11392983

# Reviewer report: Reviewer response for version 1

**DOI:** 10.21956/openreseurope.16684.r31009

**Published:** 2023-03-30

**Authors:** Tom Platteau

**Affiliations:** 1 Department of Clinical Sciences, Institute of Tropical Medicine, Antwerp, Belgium

Thank you for submitting this interesting article on the impact of ego-depletion on the intention to use condoms during sexual intercourse. This interesting, well-written manuscript presents research to assess the impact of the absence of working memory capacity / cognitive resources (ego-depletion) on sexual health behavior (intention to use condoms) via explicit and implicit associations. The authors were unable to demonstrate significant effects, and discuss potential reasons why this research question remains valid.

 Overall, the quality of the manuscript is very good. It is clear and well-written, the research question is embedded in theoretical frameworks, and the study design is adequate to answer the research question. The authors discuss sufficiently the limitations of the study, trying to explain why the hypothesis could not be demonstrated.

 I have very few general comments, and some suggestions and ideas to complement the authors’ views. Below, I list my remarks and comments. The most critical for me are (1) providing additional information on comprehensive (HIV) prevention (Introduction), and (2) potential explanation why research question is not answered (Discussion).

**Comments:**

Throughout the manuscript:

- Terminology: the authors write ‘unsafe’ or ‘safe’ sex when referring to sex without and with condoms. In recent years, there has been a shift in safer sex terminology, with having sex without a condom does not automatically implies ‘unsafe’ sex, at least for HIV (e.g., introduction of PrEP, U=U). Therefore, I suggest to write ‘condomless sex’ when referring to sex without a condom instead of ‘unsafe’ sex, and 'safer sex' instead of 'safe sex'.

Introduction:

- PrEP: related to my previous comment, I suggest to provide (brief) information on PrEP in the Introduction section. PrEP has been a gamechanger in comprehensive HIV prevention, and may impact condom use among key populations. Therefore, it is surely relevant in terms of safer sex considerations.
- In the second paragraph, the authors state: “ *This implies that when cognitive resources are sufficiently available, the explicit system will exert its influence on the behavior that is performed. It will be able to overrule the implicit system in case of conflict between both systems.*” This suggests that, if cognitive resources are available, they overrule the implicit system. Is this assumption always the case?
- When reading the last paragraph of the Introduction, the three hypotheses are repeated. This is redundant, and the paragraph may be reduced by removing repetition.

Methods:

- In the Procedure-section, I would like to read how long this procedure took for the participants. Figure 1, presenting the experimental flow, shows that the procedure is complex, but I cannot read how long it takes for the participants to complete the procedure.
- Self-control: Self-control has been manipulated via squeezing a handgrip. This is a physical task to assess a mental process (unless I am biased in my knowledge on self-control). From my perspective, self-control is a ‘cognitive process that refers to one’s ability to forego immediate or momentary pleasures, which may have negative consequences, in favor of longer-term, more abstract benefits or the prevention of adverse impacts that may never occur’ (de Ridder & de Wit, 2006 ^1^), as posited by Kotabe and Hoffman in their *integrative self-control theory* (2015 ^2^). It would be helpful (at least for me) that the authors elaborate somewhat on their concept of 'self-control' in this context.
- Tiredness: the authors describe that they used a VAS to measure participants’ tiredness. This line was ‘10mm long’. Is this correct? This is very short (only 1 centimeter).

Discussion:

- The authors propose and elaborate different reasons why the hypotheses could not be confirmed. What I missed, is a potential explanation by selecting ‘intentions to use a condom’ as outcome measure. The authors manipulated cognitive resources, and use intention to use a condom as proxy-outcome for participants’ actual behavior. However, assessing their ‘intention’ may also be a cognitive measure, instead of a behavioral. Isn’t it conceivable that ‘explicit attitudes’ dominate when assessing a cognitive measure? In other words, would it be explanatory that the authors face the ‘intention – behavior gap’, the discrepancy between peoples’ intentions (cognitive) and behavior? (Sniehotta *et al.*, 2005 ^3^; Sheeran & Webb, 2016 ^4^).

If applicable, is the statistical analysis and its interpretation appropriate?

Yes

Is the study design appropriate and is the work technically sound?

Yes

Is the work clearly and accurately presented and does it engage with the current literature?

Yes

Are the conclusions drawn adequately supported by the results?

Yes

Are sufficient details of methods and analysis provided to allow replication by others?

Yes

Are all the source data and materials underlying the results available?

Yes

Reviewer Expertise:

HIV/STI prevention, sexual health, sexualized drug use

I confirm that I have read this submission and believe that I have an appropriate level of expertise to confirm that it is of an acceptable scientific standard.

## References

[1] : Self-Regulation in Health Behavior: Concepts, Theories, and Central Issues.2006; 10.1002/9780470713150.ch1 1-23 10.1002/9780470713150.ch1

[2] : On Integrating the Components of Self-Control. *Perspect Psychol Sci* .2015;10(5) : 10.1177/1745691615593382 618-38 10.1177/1745691615593382 26386000

[3] : Bridging the intention–behaviour gap: Planning, self-efficacy, and action control in the adoption and maintenance of physical exercise. *Psychology & Health* .2005;20(2) : 10.1080/08870440512331317670 143-160 10.1080/08870440512331317670

[4] : The Intention-Behavior Gap. *Social and Personality Psychology Compass* .2016;10(9) : 10.1111/spc3.12265 503-518 10.1111/spc3.12265
